# Survivin selective inhibitor YM155 induce apoptosis in SK-NEP-1 Wilms tumor cells

**DOI:** 10.1186/1471-2407-12-619

**Published:** 2012-12-26

**Authors:** Yan-Fang Tao, Jun Lu, Xiao-Juan Du, Li-Chao Sun, Xuan Zhao, Liang Peng, Lan Cao, Pei-Fang Xiao, Li Pang, Dong Wu, Na Wang, Xing Feng, Yan-Hong Li, Jian Ni, Jian Wang, Jian Pan

**Affiliations:** 1 Department of Hematology and Oncology, Children's Hospital of Soochow University, Suzhou, China; 2 Department of Gastroenterology, the 5th Hospital of Chinese PLA, Yin chuan, Ningxia Province, China; 3 Department of Cell and Molecular Biology, Cancer Institute (Hospital), Chinese Academy of Medical Sciences, Peking Union Medical College, Beijing, China; 4 Beijing Insititute for Drug Control, Beijing, China; 5 Institute of Clinical Medical Science, China-Japan Friendship Hospital, Beijing, China; 6 Translational Research Center, Second Hospital, The Second Clinical School, Nanjing Medical University, Nanjing, China

**Keywords:** YM155, SK-NEP-1, Survivin, Apoptosis, Real-time PCR array

## Abstract

**Background:**

Survivin, a member of the family of inhibitor of apoptosis proteins, functions as a key regulator of mitosis and programmed cell death. YM155, a novel molecular targeted agent, suppresses survivin, which is overexpressed in many tumor types. The aim of this study was to determine the antitumor activity of YM155 in SK-NEP-1 cells.

**Methods:**

SK-NEP-1 cell growth *in vitro* and *in vivo* was assessed by MTT and nude mice experiments. Annexin V/propidium iodide staining followed by flow cytometric analysis was used to detect apoptosis in cell culture. Then gene expression profile of tumor cells treated with YM155 was analyzed with real-time PCR arrays. We then analyzed the expression data with MEV (Multi Experiment View) cluster software. Datasets representing genes with altered expression profile derived from cluster analyses were imported into the Ingenuity Pathway Analysis tool.

**Results:**

YM155 treatment resulted in inhibition of cell proliferation of SK-NEP-1cells in a dose-dependent manner. Annexin V assay, cell cycle, and activation of caspase-3 demonstrates that YM155 induced apoptosis in SK-NEP-1 cells. YM155 significantly inhibited growth of SK-NEP-1 xenografts (YM155 5 mg/kg: 1.45 ± 0.77 cm^3^; YM155 10 mg/kg: 0.95 ± 0.55 cm^3^) compared to DMSO group (DMSO: 3.70 ± 2.4 cm^3^) or PBS group cells (PBS: 3.78 ± 2.20 cm^3^, ANOVA P < 0.01). YM155 treatment decreased weight of tumors (YM155 5 mg/kg: 1.05 ± 0.24 g; YM155 10 mg/kg: 0.72 ± 0.17 g) compared to DMSO group (DMSO: 2.06 ± 0.38 g) or PBS group cells (PBS: 2.36 ± 0.43 g, ANOVA P < 0.01). Real-time PCR array analysis showed between Test group and control group there are 32 genes significantly up-regulated and 54 genes were significantly down-regulated after YM155 treatment. Ingenuity pathway analysis (IPA) showed cell death was the highest rated network with 65 focus molecules and the significance score of 44. The IPA analysis also groups the differentially expressed genes into biological mechanisms that are related to cell death, cellular function maintenance, cell morphology, carbohydrate metabolism and cellular growth and proliferation. Death receptor signaling (3.87E-19), TNFR1 signaling, induction of apoptosis by HIV1, apoptosis signaling and molecular mechanisms of cancer came out to be the top four most significant pathways. IPA analysis also showed top molecules up-regulated were BBC3, BIRC3, BIRC8, BNIP1, CASP7, CASP9, CD5, CDKN1A, CEBPG and COL4A3, top molecules down-regulated were ZNF443, UTP11L, TP73, TNFSF10, TNFRSF1B, TNFRSF25, TIAF1, STK17A, SST and SPP1, upstream regulator were NR3C1, TP53, dexamethasone , TNF and Akt.

**Conclusions:**

The present study demonstrates that YM155 treatment resulted in apoptosis and inhibition of cell proliferation of SK-NEP-1cells. YM155 had significant role and little side effect in the treatment of SK-NEP-1 xenograft tumors. Real-time PCR array analysis firstly showed expression profile of genes dyes-regulated after YM155 treatment. IPA analysis also represents new molecule mechanism of YM155 treatment, such as NR3C1 and dexamethasone may be new target of YM155. And our results may provide new clues of molecular mechanism of apoptosis induced by YM155.

## Background

Wilms tumor (WT) is the most common malignant neoplasm of the urinary tract in children [1]. Although it is curable with long-term survival, the combination of surgery, chemotherapy and often radiotherapy in some cases results in severe complications in adulthood [2]. Therefore, novel therapeutic strategies that would decrease treatment burden and improve outcome for high risk patients are required. We evaluated the efficacy of YM155, an inhibitor of survivin, to inhibit Wilms tumor development in xenografts models.

Overexpressed survivin can be detected in virtually every human tumor, but undetectable or present at very low levels in most normal adult tissues [3-5]. A ‘tumor-specific’ expression of survivin is predominantly dictated at the level of transcription, and that survivin gene expression may be globally ‘deregulated’ in tumors, *in vivo*[4,6,7]. Accordingly, survivin promoter activity is basically silent in normal cells, but strongly activated in tumor cells, and this occurs independently of cellular heterogeneity, mitotic status, or genetic makeup. The differential expression of the survivin gene in normal versus tumor cells is so dramatic that therapeutic strategies to drive tumor-specific expression of suicidal genes under the control of the survivin promoter have now advanced to preclinical stages in a number of settings [3,5-9].

YM155, a novel small-molecule survivin suppressant, has been shown to suppress survivin with little effect on expression levels of other IAP family or Bcl-2 related proteins [10]. YM155 has been demonstrated antitumor activity, with survivin suppression and tumor cell apoptosis, in various human cancer models [6,8,10-17]. Survivin is the smallest member of the Inhibitor of Apoptosis (IAP) gene family. Originally described as cell survival factors that target caspase, we now know that IAPs have a much broader portfolio of functions, encompassing signaling pathways, cell division, metabolism and adaptation to unfavorable environments. Survivin embodies this multifunctional diversity, and compelling data accumulated over a decade have elucidated many of its essential roles as a regulator of mitosis [17], a broad cytoprotective factor [18,19], and an effector of cellular adaptation to stress [20,21]. These disparate functions rely on hosts of regulated interactions that involve survivin and multiple protein partners, including tubulin [22,23] and various chromosomal passenger proteins in the control of mitosis, other IAP family members to counteract apoptosis, and Heat Shock Proteins in the modulation of the cellular stress response. These ‘survivin networks’ are dramatically exploited in cancer, and survivin is unanimously viewed as one of the most prominent cancer genes.

In the present study, the antitumor effect of YM155 have been evaluated in human SK-NEP-1 Wilms tumor cells and xenograft models to further characterize its preclinical efficacy, and the molecular mechanism was exploited with real-time PCR arrays.

## Methods

### Cell and culture conditions

SK-NEP-1 Human kidney (Wilm's Tumor) cell line obtained from the American Type Culture Collection (ATCC) was maintained in the Maccyo’5 (Life Technologies Inc., Gaithersburg, MD, USA) supplemented with 20% heat-inactivated fetal bovine serum (Invitrogen Co., NY, USA) in a humidified incubator with 5% CO_2_ at 37°C. YM155 (Cat: S1130 Selleck Chemicals, West Paterson, NJ, USA) was dissolved in DMSO (Cat: D4540 Sigma–Aldrich, St. Louis, MO, USA)

### Cell proliferation

Sk-NEP-1 cells (2×10^4^) were seeded in 96-well plates overnight and incubated with DMSO, 1 nM YM155, or increasing concentrations of YM155 (0.005, 0.01, 0.02, 0.04, 0.08, 0.16, 0.32, 0.64 or 1.28 μM) for 24 hours. The volume of DMSO added to the vehicle treated wells was the same as that added to the drug treated wells. Each drug concentration was performed in four replicate wells. 20uLMTT (3-(4, 5-dimethylthiazol-2-yl)-2, 5- diphenyltetrazolium bromide) solution (5 mg/ml) was added to each well and incubated at 37°C for a further 4 hours. Then 200 uL of DMSO was added to each well after the medium was removed. The optical density (OD) values were measured at 490 nm on a scanning multi-well spectrophotometer (BioRad Model 550, USA). Compared with the control group, the relative survival rate of remained cells was calculated from the absorbance values. Cell proliferation was calculated as a percentage of the DMSO- treated control wells with IC50 values derived after plotting proliferation values on a logarithmic curve.

### Cell cycle analysis

Cells were collected and washed with PBS for 5 minutes by centrifugation at 125 × g. Cells were fixed with paraformaldehyde and transparented with 0.5% Triton X-100. Then cells were resuspended in a staining solution containing 1.5 μmol/L propidium iodide (P4170, Sigma–Aldrich, St. Louis, MO, USA) and 25 μg/ml RNase A and incubated for 30 minutes in 37°C. The samples (10000 cells) were analyzed by fluorescence-activated cell sorting with a Beckman Gallios™ Flow Cytometer.

### Apoptosis assay

Apoptosis assay was according to the manual operation of BD Annexin V Staining Kit (Cat: 556420, BD Biosciences, Franklin Lakes, NJ USA). Briefly, wash cells twice with cold PBS and then resuspend cells in 1×Binding Buffer at a concentration of ~1 ×10^6^ cells/ml. Transfer 100 μl of the solution (~1×10^5^ cells) to a 5 ml culture tube. Add Annexin V and PI 5 μl/test. Gently mix the cells and incubate for 15 min at RT in the dark. Add 400 μl of 1×Binding Buffer to each tube. Analyze by flow cytometry as soon as possible (within 1 hour).

### Western blot analysis

For western blot analysis, cellular proteins were extracted in 40 mM Tris–HCl (pH 7.4) containing 150 mM NaCl and 1% (v/v) Triton X-100, supplemented with a cocktail of protease inhibitors. Equal amounts of protein were resolved on 12% SDS-PAGE gels, and then transferred to a PVDF membrane (Millipore, Bedford, MA). Blots were blocked and then probed with antibodies against Caspase 3 (1:1000,

Cell Signaling Technology, Inc. Danvers, MA), GAPDH (1:5000, Sigma, St. Louis, MO). After washing, the blots were incubated with horseradish peroxidase-conjugated secondary antibodies and visualized by enhanced chemiluminescence kit (Pierce, Rockford, IL). Protein bands were visualized after exposure of the membrane to Kodak X-ray film.

### Xenograft assays the treatment effect of YM155 in nude mice

This study was carried out in strict accordance with the recommendations in the Guide for the Care and Use of Laboratory Animals of the National Institutes of Health. The protocol was approved by the Committee on the Ethics of Animal Experiments of Soochow university (Permit Number: 2011-10-21). All surgery was performed under sodium pentobarbital anesthesia, and all efforts were made to minimize suffering. Female nu/nu mice, aged 4–6 weeks, obtained from the Jackson Laboratory (Vitalriver, China), were kept in Class 10000 Clean Room at the Laboratory Animal Center of Soochow University (http://dwzx.suda.edu.cn/pages/index.aspx.) SK-NEP-1 cells were subcutaneously injected into five nude mice every group. 10 days after injection, mice were treated with PBS, DMSO, YM155 5 mg/kg and 10 mg/kg dose. During the next six weeks these mice were examined for subcutaneous tumor growth. The tumor volumes were calculated according to the following formula: volume = length × width^2^/2. After the last treatment, the mice were killed and the tumor weight was measured.

### Real-time PCR array analysis

For RNA extraction, cells were immediately submerged in 2 ml Trizol (Invitrogen Co., NY, USA), stored at −80°C until further processed. A volume of 1 ml of each sample was spun at 4°C for 15 min at 12,000 g to remove debris and DNA, 1 ml of supernatant was mixed with 200 ul chloroform, shaken for 15 seconds, incubated at Room Temperature for 2–3 minutes and spun for 10 minutes at 12,000 g at 4°C. RNA was precipitated by adding 500 ul of the aqueous phase to an equal volume of isopropanol and spun at 14,000 g at 4°C for 10 minutes. RNA was washed with 75% ethanol, spun at 14,000 g at 4°C for 10 minutes, dried and resuspended in 40 ul DEPC-treated H_2_O . The final RNA concentration was determined using a spectrophotometer (Nanodrop 2000) and the purity was assessed by agarose gel electrophoresis. CDNA synthesis was performed on 4 ug of RNA in a 10 ul sample volume using SuperScript II reverse transcriptase (Invitrogen Co., NY, USA) as recommended by the manufacturer. The RNA was incubated with 0.5 ug of oligo(dT)12–18mers primers (Invitrogen Co., NY, USA) for 7 minutes at 70°C and then transferred onto ice. Then, 9 ul of a master mix containing 4 ul of SuperScript II buffer, 2 ul of 0.1 M DTT, and 1 ul each of dNTPs stock (10 mM), Rnasin (40 UI) and SuperScript II were added to the RNA sample, spun and incubated at 42°C for 60 min followed by 5 min at 70°C to inactivate the enzyme. CDNA was stored at −20°C. Real-time PCR array (SABioscience Human Apoptosis PCR Array PAHS-3012) analysis was performed in a total volume of 20 ul including 2ul of cDNA, primers (0.2 mM each) and 10 ul of SYBR Green mix (Roche Co., Basel, Switzerland.). Reactions were run on an Light cycler 480 using the universal thermal cycling parameters (95°C 5 min, 45 cycles of 10 sec at 95°C, 20 sec at 60°C and 15 sec at 72°C; melting curve: 10 sec at 95°C, 60 sec at 60°C and continues melting). Results were obtained using the sequence detection software Light cycler 480 and analyzed using Microsoft Excel. For all samples melting curves were acquired for quality control purposes. For gene expression quantification, we used the comparative Ct method. First, gene expression levels for each sample were normalized to the expression level of the housekeeping gene encoding Glyceraldehydes 3-phosphate dehydrogenase (GAPDH) within a given sample (−ΔCt); the relative expression of each gene was calculated with10^6^ *Log_2_(−ΔCt ).The difference between the YM155 treatment samples compared to the control samples was used to determine the10^6^ *Log_2_(−ΔCt ). Statistical significance of the gene expression difference between the YM155 treatment and the control samples was calculated with the T-test using SPSS 11.5 software.

### Ingenuity pathway analysis (IPA)

Datasets representing genes with altered expression profile derived from Real-time PCR array analyses were imported into the Ingenuity Pathway Analysis Tool (IPA Tool; Ingenuity H Systems, Redwood City, CA, USA; http://www.ingenuity.com). In IPA, differentially expressed genes are mapped to genetic networks available in the Ingenuity database and then ranked by score. The basis of the IPA program consists of the Ingenuity Pathway Knowledge Base (IPKB) which is derived from known functions and interactions of genes published in the literature. Thus, the IPA Tool allows the identification of biological networks, global functions and functional pathways of a particular dataset. The program also gives the significance value of the genes, the other genes with which it interacts, and how the products of the genes directly or indirectly act on each other, including those not involved in the microarray analysis. The networks created are ranked depending on the number of significantly expressed genes they contain and also list diseases that were most significant. A network is a graphical representation of the molecular relationships between molecules. Molecules are represented as nodes, and the biological relationship between two nodes is represented as an edge (line). All edges are supported by at least 1 reference from the literature, from a textbook, or from canonical information stored in the Ingenuity Pathways Knowledge Base.

### Statistical analysis

At least three replicates for each experimental condition were performed, and the presented results were representative of these replicates. All values are presented as means ± SEM. Student’s paired t-test was applied to reveal statistical significances. P values less than 0.05 were considered significant. Statistical analyses were performed using SPSS Software for Windows (version 11.5; SPSS, Inc., Chicago, IL).

## Results

### Growth inhibitory effect of YM155 on SK-NEP-1 cells

**Figure 1:**
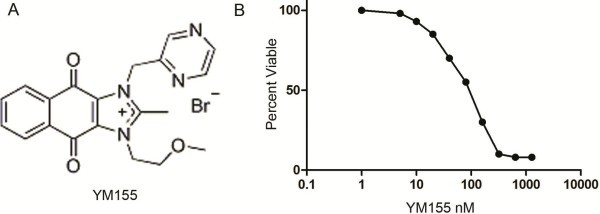
**Growth inhibitory effect of YM155 on SK-NEP-1 cells. **(**A**) Molecular Structure of YM155. (**B**) Proliferation and IC50 analysis of YM155. Sk-NEP-1 cells (2 × 10^4^) were seeded in 96-well plates overnight and incubated with DMSO, 1 nM YM155, or increasing concentrations of YM155 (0.005, 0.01, 0.02, 0.04, 0.08, 0.16, 0.32, 0.64or 1.28 μM) for 24 hours. Compared with the control group, the relative survival rate of remained cells was calculated from the absorbance values. Cell proliferation was calculated as a percentage of the DMSO treated control wells with IC50 values derived after plotting proliferation values on a logarithmic curve. Experiments were performed in quadruplicate and repeated two times.

YM155 treatment resulted in inhibition of cell proliferation of SK-NEP-1cells in a dose-dependent manner (Figure 1B). 93%, 85%, 70%, 55% and 30% cells were viable when 10nM, 20nM, 40nM, 80nM and 160nM YM155 treated 24 hours. So the IC50 of YM155 for SK-NEP-1 cells was about 100nM. The adherence of SK-NEP-1 cells was much inhibited by YM155-treatment. YM155 induced detachment of the cells from the dishes (Data not shown).

### YM155 induced apoptosis in SK-NEP-1 cells

**Figure 2:**
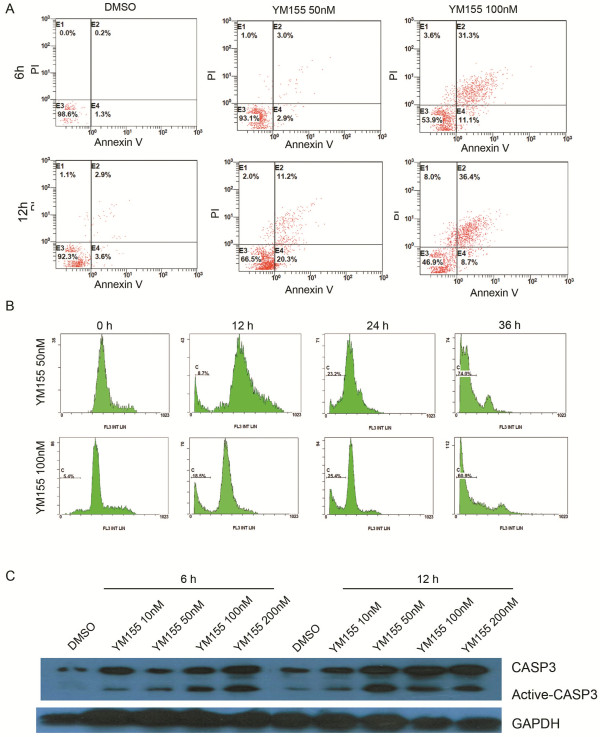
**YM155 induced apoptosis in SK-NEP-1 cells. **YM155 induced apoptosis, DNA fragmentation and activation of caspase 3 in SK-NEP-1 cells. (**A**) Annexin V analysis showed the cells treated with YM155 50nM and 100nM for 6 and 12 hours, much more cells showed apoptotic feature compared with control group. These analyses were repeated three times, and the apoptotic cells in 6 hours was YM155 50nM: 5.9% ±2.3%,YM155 100nM 42.4% ±8.9% compared to DMSO group: 1.5%±0.4%; apoptotic cells in 12 hours was YM155 50nM: 31.5% ±5.7%, YM155 100nM 45.1% ±11.3% compared to DMSO group 6.5% ±2.1%. (**B**) Cell cycle analysis the cells treated with YM155 after 12, 24 and 36 hours. As expected, DNA fragmentation was observed after 12 hours treatment and increased in a time dependent manner. These analyses were repeated three times, and the apoptotic cells in YM155 50nM group: 12 hours 8.7% ±1.4%, 24 hours 23.2% ±6.7%, 36 hours 74.0%±9.2%; apoptotic cells in YM155 100nM group: 12 hours 18.5% ±3.7% , 24 hours 25.4% ±9.2% , 36 hours 60.9% ±14.2%. (**C**) Western-blot analysis the activation of caspase 3 in cells treated with YM155. After 6 and 12 hours treatment with 10nM, 50nM, 100nM and 200nM YM155, cleaved caspase-3 was observed.

To confirm whether YM155 induces apoptosis in SK-NEP-1 cells, we further investigated Annexin V assay, cell cycle, and activation of caspase-3 in SK-NEP-1 cells after YM155 treatment. The result showed that cells treated with YM155 50nM and 100nM for 6 and 12 hours, much more cells showed apoptotic feature compared with control group (Figure 2A). These analyses were repeated three times, and the apoptotic cells in 6 hours was YM155 50nM: 5.9% ±2.3%,YM155 100nM 42.4% ±8.9% compared to DMSO group: 1.5%±0.4%; apoptotic cells in 12 hours was YM155 50nM: 31.5% ±5.7%, YM155 100nM 45.1% ±11.3% compared to DMSO group 6.5% ±2.1%. Cell cycle was confirmed by Cell cycle assay (Figure 2B). As expected, DNA fragmentation was observed after 12 hours treatment and increased in a time dependent manner. These analyses were repeated three times, and the apoptotic cells in YM155 50nM group: 12 hours 8.7% ±1.4%, 24 hours 23.2% ±6.7%, 36 hours 74.0%±9.2%; apoptotic cells in YM155 100nM group: 12 hours 18.5% ±3.7% , 24 hours 25.4% ±9.2% , 36 hours 60.9% ±14.2%. Moreover, to clearly demonstrate that YM155 causes apoptosis in SK-NEP-1 cells, we assessed the molecular aspects of apoptosis, caspase-3, well recognized as a marker of apoptosis by western blot. After 6 and 12 hours treatment with 10nM, 50nM, 100nM and 200nM YM155, cleaved caspase-3 was observed (Figure 2C). This result is consistent with the data of Annexin V assay and cell cycle analysis, demonstrating that YM155 induced apoptosis in SK-NEP-1 cells. The results from both studies suggest that YM155 has promising antitumor activity against SK-NEP-1 cells.

### YM155 treatment inhibited growth of SK-NEP-1 xenograft tumor in nude mice

**Figure 3:**
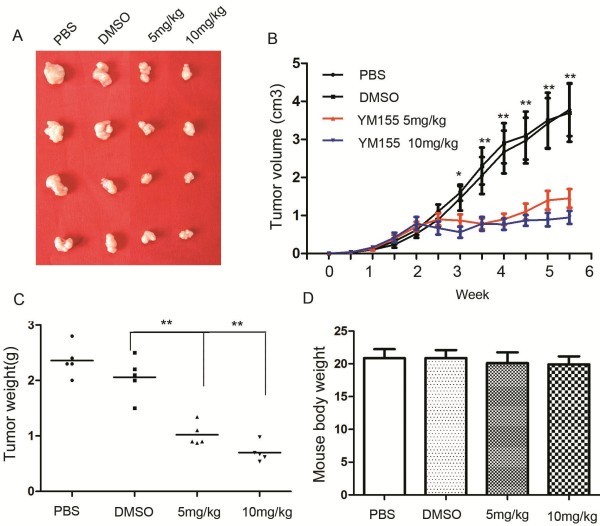
**YM155 treatment inhibited growth of SK-NEP-1 xenograft tumor in nude mice. **SK-NEP-1 cells were subcutaneously injected into five nude mice every group. 10 days after injection, mice were treated with PBS, DMSO, YM155 5 mg/kg and 10 mg/kg dose. During the next six weeks these mice were examined for subcutaneous tumor growth. The tumor volumes were calculated according to the following formula: volume = length × width^2^/2. After the last treatment, the mice were killed and the tumor weight was measured. (**A**) SK-NEP-1 xenograft tumors from the treatment experiment. (**B**) Growth curve of SK-NEP-1 cells treated with YM155, DMSO and PBS. YM155 significantly inhibited growth of SK-NEP-1 xenografts (YM155 5 mg/kg: 1.45 ± 0.77 cm^3^; YM155 10 mg/kg: 0.95 ± 0.55 cm^3^) compared to DMSO group (DMSO: 3.70 ± 2.4 cm^3^) or PBS group cells (PBS: 3.78 ± 2.20 cm^3^, ANOVA P < 0.01). (**C**) Tumor weight of the treatment experiment. YM155 treatment decreased weight of tumors (YM155 5 mg/kg: 1.05 ± 0.24 g; YM155 10 mg/kg: 0.72 ± 0.17 g) compared to DMSO group (DMSO: 2.06 ± 0.38 g) or PBS group cells (PBS: 2.36 ± 0.43 g, ANOVA P < 0.01). (**D**) Body weight of nude mice in the treatment experiment. Body weight of nude mice treated with YM155 was almost same with control group (YM155 5 mg/kg:20.12 ± 1.66 g; YM155 10 mg/kg: 19.92 ± 1.72 g) compared to DMSO group (DMSO: 20.88 ± 1.83 g) or PBS group cells (PBS: 20.66 ± 1.37 g, ANOVA P >0.05).

We next assessed the impact of YM155 on the cell growth of SK-NEP-1 cells in nude mice. SK-NEP-1 cells were subcutaneously injected into five nude mice every group. 10 days after injection, mice were treated with PBS, DMSO, YM155 5 mg/kg and 10 mg/kg dose. During the next six weeks these mice were examined for subcutaneous tumor growth. After the last treatment, the mice were killed and the tumor weight was measured. YM155 significantly inhibited growth of SK-NEP-1 xenografts (YM155 5 mg/kg: 1.45 ± 0.77 cm^3^; YM155 10 mg/kg: 0.95 ± 0.55 cm^3^) compared to DMSO group (DMSO: 3.70 ± 2.4 cm^3^) or PBS group cells (PBS: 3.78 ± 2.20 cm^3^, ANOVA P < 0.01 Figure 3A, 3B). YM155 treatment decreased weight of tumors (YM155 5 mg/kg: 1.05 ± 0.24 g; YM155 10 mg/kg: 0.72 ± 0.17 g) compared to DMSO group (DMSO: 2.06 ± 0.38 g) or PBS group cells (PBS: 2.36 ± 0.43 g, ANOVA P < 0.01 Figure 3C). We also observed that the body weight of nude mice treated with YM155 was almost same with control group (YM155 5 mg/kg:20.12 ± 1.66 g; YM155 10 mg/kg: 19.92 ± 1.72 g) compared to DMSO group (DMSO: 20.88 ± 1.83 g) or PBS group cells (PBS: 20.66 ± 1.37 g, ANOVA P >0.05 Figure 3D). These studies support the view that YM155 had significant role and little side effect in the treatment of SK-NEP-1 xenograft tumors.

### Real-time PCR array analysis the dyes-regulated genes implicated into YM155 treatment

In order to identify apoptosis and/or programmed cell death molecules implicated into the treatment with YM155, we used the SABioscience Human Apoptosis PCR Array PAHS-3012.We analyzed and clustered the expression of 370 key genes involved in apoptosis, or programmed cell death with this PCR Array (Additional file 1). This array includes the TNF ligands and their receptors; members of the bcl-2 family, BIRC (baculoviral IAP repeat) domain proteins, CARD domain (caspase recruitment domain) proteins, death domain proteins, TRAF (TNF receptor-associated factor) domain proteins and caspases. We can easily and reliably analyze the expression of a focused panel of genes related to apoptosis with this array. Comparison of PCR results between Test group and control group showed that 32 genes were significantly up-regulated and 54 genes were significantly down-regulated after YM155 treatment (Table 1 and Table 2).

**Table 1:** Genes up regulated in SK-NEP-1 cells treated with YM155 compared with DMSO control group

	**Gene**	**Symbol**	**+DMSO**	**+YM155**	**Ratio**	**P value**
1	TNF	Tumor necrosis factor	0.052	3.274	62.850	0.0085
2	FOXO1	Forkhead box O1	3.109	59.967	19.287	0.0091
3	IER3	Immediate early response 3	53.221	795.891	14.955	0.0093
4	PEA15	Phosphoprotein enriched in astrocytes 15	85.200	642.789	7.545	0.0105
5	CD5	CD5 molecule	0.079	0.584	7.405	0.0105
6	NDUFS3	NADH dehydrogenase (ubiquinone) Fe-S protein 3,	127.186	919.431	7.229	0.0106
7	TNFAIP3	Tumor necrosis factor, alpha-induced protein 3	69.132	449.671	6.505	0.0109
8	NFKB1	nuclear factor of kappa gene enhancer in B-cells 1	58.200	377.771	6.491	0.0109
9	CRYAB	Crystallin, alpha B	7.704	49.315	6.401	0.0109
10	DDIT3	DNA-damage-inducible transcript 3	134.710	768.681	5.706	0.0113
11	CRADD	CASP2 domain containing adaptor with death domain	10.127	56.007	5.531	0.0114
12	BNIP1	BCL2/adenovirus E1B 19 kDa interacting protein 1	89.770	475.609	5.298	0.0115
13	BBC3	BCL2 binding component 3	1.388	6.896	4.969	0.0118
14	NOX5	NADPH oxidase, EF-hand calcium binding domain 5	0.213	1.057	4.953	0.0118
15	CASP7	Caspase 7, apoptosis-related cysteine peptidase	20.274	95.136	4.693	0.0120
16	PIM2	Pim-2 oncogene	29.093	118.888	4.087	0.0127
17	CEBPG	CCAAT/enhancer binding protein (C/EBP), gamma	11.867	47.745	4.023	0.0128
18	EDA	Ectodysplasin A	0.710	2.846	4.009	0.0128
19	SOCS2	Suppressor of cytokine signaling 2	0.258	1.018	3.953	0.0129
20	CDKN1A	Cyclin-dependent kinase inhibitor 1A (p21, Cip1)	124.431	479.383	3.853	0.0131
21	IL1A	Interleukin 1, alpha	146.968	423.629	2.882	0.0155
22	BIRC8	Baculoviral IAP repeat containing 8	0.248	0.692	2.785	0.0159
23	SERPINB2	Serpin peptidase inhibitor, clade B member 2	0.136	0.362	2.659	0.0165
24	CASP9	Caspase 9, apoptosis-related cysteine peptidase	330.442	853.550	2.583	0.0169
25	LTB	Lymphotoxin beta (TNF superfamily, member 3)	0.052	0.134	2.580	0.0170
26	PMAIP1	Phorbol-12-myristate-13-acetate-induced protein 1	258.441	642.950	2.488	0.0175
27	NUPR1	Nuclear protein, transcriptional regulator, 1	25.295	61.632	2.437	0.0179
28	BIRC3	Baculoviral IAP repeat containing 3	97.141	232.642	2.395	0.0183
29	DIABLO	IAP-binding mitochondrial protein	178.419	417.832	2.342	0.0187
30	LTBR	Lymphotoxin beta receptor	120.546	280.744	2.329	0.0188
31	COL4A3	Collagen, type IV, alpha 3	5.993	12.916	2.155	0.0208
32	FOXO3	Forkhead box O3	34.671	71.488	2.062	0.0222

**Table 2:** Genes down regulated in SK-NEP-1 cells treated with YM155 compared with DMSO control group

	**Gene**	**Symbol**	**+DMSO**	**+YM155**	**Ratio**	**P value**
1	HIPK2	Homeodomain interacting protein kinase 2	31.640	11.619	0.367	0.0353
2	CD27	CD27 molecule	1.462	0.538	0.368	0.0353
3	PCBP4	Poly(rC) binding protein 4	9.419	3.481	0.370	0.0334
4	PAK7	P21 protein (Cdc42/Rac)-activated kinase 7	0.120	0.044	0.370	0.0334
5	PPP1R13B	Protein phosphatase 1, regulatory subunit 13B	75.280	27.926	0.371	0.0322
6	TP73	Tumor protein p73	9.738	3.619	0.372	0.0321
7	RASA1	RAS p21 protein activator 1	138.406	49.465	0.357	0.0315
8	CRYAA	Crystallin, alpha A	0.210	0.079	0.375	0.0272
9	TIAF1	TGFB1-induced anti-apoptotic factor 1	243.057	91.076	0.375	0.0242
10	CARD14	Caspase recruitment domain family, member 14	37.791	13.412	0.355	0.0241
11	NOD1	Nucleotide-binding domain containing 1	163.081	61.858	0.379	0.0214
12	TNFRSF1B	Tumor necrosis factor receptor superfamily 1B	45.110	17.323	0.384	0.0174
13	HIP1	Huntingtin interacting protein 1	14.568	4.941	0.339	0.0174
14	UTP11L	UTP11-like, U3 small nucleolar ribonucleoprotein	267.624	104.245	0.390	0.0118
15	SPP1	Secreted phosphoprotein 1	2.389	0.801	0.335	0.0107
16	NME5	NME/NM23 family member 5	0.217	0.073	0.335	0.0092
17	MYO18A	Myosin XVIIIA	164.894	54.821	0.332	0.0087
18	PPP2R1B	Protein phosphatase 2, regulatory subunit A	9.768	3.887	0.398	0.0033
19	BAG1	BCL2-associated athanogene	70.273	28.001	0.398	0.0033
20	DAPK2	Death-associated protein kinase 2	1.549	0.509	0.329	0.0023
21	TNFSF10	**T**umor necrosis factor (ligand) superfamily 10	2.596	0.844	0.325	0.0022
22	TNFRSF25	Tumor necrosis factor receptor superfamily 25	10.685	4.340	0.406	0.0022
23	FOXL2	Forkhead box L2	11.954	4.886	0.409	0.0021
24	CASP10	Caspase 10, apoptosis-related cysteine peptidase	13.465	4.252	0.316	0.0021
25	CARD8	Caspase recruitment domain family, member 8	3.835	1.209	0.315	0.0020
26	ALOX12	Arachidonate 12-lipoxygenase	9.725	3.039	0.312	0.0020
27	OPA1	Optic atrophy 1 (autosomal dominant)	172.500	71.720	0.416	0.0020
28	ERN2	Endoplasmic reticulum to nucleus signaling 2	2.851	1.187	0.416	0.0020
29	STK17A	Serine/threonine kinase 17a	111.835	46.583	0.417	0.0020
30	CARD9	Caspase recruitment domain family, member 9	5.195	1.578	0.304	0.0020
31	GRM4	Glutamate receptor, metabotropic 4	0.958	0.408	0.426	0.0020
32	SON	SON DNA binding protein	547.934	234.409	0.428	0.0019
33	PLAGL2	Pleiomorphic adenoma gene-like 2	69.972	30.027	0.429	0.0019
34	BIRC5	Baculoviral IAP repeat containing 5	59.315	25.502	0.430	0.0019
35	FADD	Fas (TNFRSF6)-associated via death domain	323.082	93.703	0.290	0.0019
36	NAIP	NLR family, apoptosis inhibitory protein	44.019	12.731	0.289	0.0008
37	BCL2	B-cell CLL/lymphoma 2	6.950	3.020	0.435	0.0008
38	ZNF443	Zinc finger protein 443	8.149	3.583	0.440	0.0003
39	CASP8AP2	**C**aspase 8 associated protein 2	43.673	19.445	0.445	0.0003
40	CUL5	Cullin 5	134.969	60.117	0.445	0.0002
41	DAPK1	Death-associated protein kinase 1	15.806	4.237	0.268	<0.0001
42	NKX3-2	NK3 homeobox 2	0.830	0.371	0.447	<0.0001
43	SEMA4D	Itransmembrane domain semaphoring 4D	9.709	2.589	0.267	<0.0001
44	SST	Somatostatin	0.694	0.179	0.258	<0.0001
45	ALOX15B	Arachidonate 15-lipoxygenase, type B	0.263	0.063	0.242	<0.0001
46	NOTCH2	Notch 2	930.886	223.933	0.241	<0.0001
47	NLRC4	NLR family, CARD domain containing 4	20.624	4.653	0.226	<0.0001
48	PRKCA	Protein kinase C, alpha	30.560	6.439	0.211	<0.0001
49	APOE	Apolipoprotein E	1.075	0.210	0.195	<0.0001
50	NTF3	Neurotrophin 3	0.295	0.044	0.150	<0.0001
51	CARD17	Caspase recruitment domain family, member 17	0.363	0.047	0.131	<0.0001
52	MAPK8IP2	Mitogen kinase 8 interacting protein 2	0.432	0.049	0.114	<0.0001
53	F2	coagulation factor II	3.794	0.104	0.027	<0.0001
54	CUL3	cullin 3	1.228	0.059	0.048	<0.0001

### Ingenuity pathway analysis the pathway regulated by YM155

**Figure 4:**
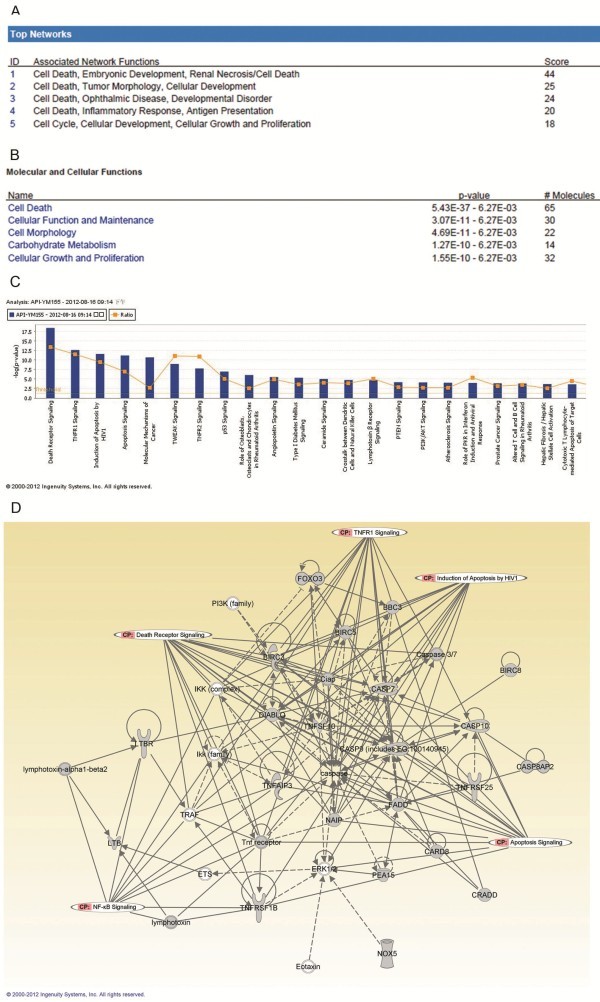
**Ingenuity Pathways Analysis (IPA) summary. **To investigate possible interactions of differently regulated genes, datasets representing 86 genes with altered expression profile obtained from real-time PCR arrays were imported into the Ingenuity Pathway Analysis Tool and the following data is illustrated: (**A**) The list of top five networks with their respective scores obtained from IPA . (**B**) The list of top five molecular and cellular functions with their respective scores obtained from IPA. (**C**) Toxicology pathway list in IPA analysis. The x-axis represents the top toxicology functions as calculated by IPA based on differentially expressed genes are highlighted and the y-axis represents the ratio of number of genes from the dataset that map to the pathway and the number of all known genes ascribed to the pathway. The yellow line represents the threshold of p value, 0.05 as calculated by Fischer’s test. (**D**) Most highly rated network in IPA analysis. The network representation of the most highly rated network. The genes that are shaded were determined to be significant from the statistical analysis. A solid line represents a direct interaction between the two gene products and a dotted line means there is an indirect interaction.

To investigate possible biological interactions of differently regulated genes, datasets representing genes with altered expression profile derived from real-time PCR array analyses were imported into the Ingenuity Pathway Analysis Tool. The list of differentially expressed genes analyzed by IPA revealed significant networks. Figure 4A represents the list of top 5 networks identified by IPA. Of these networks, cell death was the highest rated network with 65 focus molecules and the significance score of 44 (Figure 4D). The score is the probability that a collection of genes equal to or greater than the number in a network could be achieved by chance alone. A score of 3 indicates a 1/1000 chance that the focus genes are in a network not due to random chance. The IPA analysis also groups the differentially expressed genes into biological mechanisms that are related to cell death, cellular function maintenance, cell morphology, carbohydrate metabolism and cellular growth and proliferation (Figure 4B). Death receptor signaling (3.87E-19), TNFR1 signaling (2.34E-13), induction of apoptosis by HIV1 (2.67E-12), apoptosis signaling (6.56E-12) and molecular mechanisms of cancer (2.13E-11) came out to be the top four most significant pathways (Figure 4C). IPA analysis also showed top molecules up-regulated were BBC3,BIRC3,BIRC8,BNIP1,CASP7,CASP9,CD5,CDKN1A,CEBPG and COL4A3, top molecules down-regulated were ZNF443, UTP11L, TP73, TNFSF10, TNFRSF1B, TNFRSF25,TIAF1,STK17A,SST and SPP1, upstream regulator were NR3C1, TP53, dexamethasone , TNF and Akt ( Additional file 2). These upstream regulators such as TP53, TNF and Akt have already been reported as important regulators for the surviving network. TP53 and Akt have been widely investigated and there are hundreds of papers about the important roles in surviving pathway. But there is still no report about the relationship between NR3C1, dexamethasone and survivin.

This work indicated firstly that NR3C1and dexamethasone may be upstream regulators in the survivin pathway. These results may provide new clues of molecular mechanism of apoptosis induced by YM155.

## Discussion

Survivin is highly expressed in a broad range of solid tumors and hematological malignancies. Increased survivin expression in cancer patients is an unfavorable prognostic marker correlating with decreased overall survival in several malignancies, including non-small cell lung [24-26], gastric [27-31], colorectal [32-34], and breast carcinomas [35], neuroblastoma [36], prostate cancer [37], pancreatic cancer [38] , hepatocellular carcinoma [39] and hematologic malignancies [40-44]. Increased survivin expression was also associated with increased risk of recurrence, lymph node invasion and metastasis. Finally, survivin overexpression may be a predictive factor to determine response to chemotherapy and radiotherapy in patients with bladder cancer, breast cancer, multiple myeloma and lymphoma. Studies have shown that survivin suppression induces tumor cell apoptosis and enhances sensitivity to apoptosis induced by existing anticancer drugs and other apoptotic stimuli. This work indicated that survivin also be an important target for human Wilms tumor cells.

YM155 is a novel survivin suppressant that is currently in clinical development by Astellas Pharma, Inc. YM155 inhibited the growth of 119 human cancer cell lines, with the greatest activity in lines derived from non-Hodgkin’s lymphoma, hormone-refractory prostate cancer, ovarian cancer, sarcoma, non-small-cell lung cancer, breast cancer, leukemia and melanoma. The mean log growth inhibition of 50% (GI50) value was 15 nM. A preclinical study showed that YM155 suppressed both survivin protein and mRNA expression. In a toxicologic study, short-term exposure at high blood concentrations caused cardiotoxicity in the form of atrioventricular. In this phase I study, YM155 seemed to be safe and well-tolerated, with a maximum tolerated dose of 8.0 mg/m^2^/d. Stable disease was achieved in nine patients. The data in this study indicate that the adverse reactions observed can be well-controlled by taking due caution and suggest that YM155 has more easily controllable toxicities compared with conventional cytotoxic anticancer drugs. This work also supports the view that YM155 had significant role and little side effect in the treatment of SK-NEP-1 xenograft tumors.

Real-time PCR Array System is the ideal tool for analyzing the expression of a focused panel of genes. The flexibility, simplicity, and convenience of standard SYBR Green PCR detection methodology make the PCR Array System accessible for routine use in any research laboratory [45]. In this study, we analyzed the dyes-regulated genes by YM155 with this powerful platform, Real-time PCR arrays.

Comparison of PCR results between Test group and control group showed that 32 genes were significantly up-regulated and 54 genes were significantly down-regulated after YM155 treatment. Some genes, such as TNF, NFKB1, CDKN1A, CASP9, COL4A3, BIRC5 (survivin), BCL2, and DAPK1 have already been reported with YM155 treatment. There are also some other genes never reported with YM155 treatment and these genes have complicate functions far exceeds the apoptosis. These results consistent with the complicate roles of survivin in cancer biology. Survivin has been implicated in the regulation of the mitotic spindle checkpoint, from kinetic core to spindle assembly; it’s over expression in cancer may allow cells with spindle defects or misaligned kinetic cores to continue through cell division. Recent studies also suggest that survivin plays a role in tumor progression and chemoresistance. Survivin has been shown to inhibit cell death induced by several anticancer agents, including paclitaxel [46], etoposide [47] and Tumor Necrosis Factor-a related apoptosis-inducing ligand [47,48]. In vitro and in vivo studies showed that inhibiting survivin reduces tumor growth potential and sensitizes tumor cells to chemotherapeutic agents, such paclitaxel, cisplatin [14,49], etoposide, gamma irradiation and immunotherapy. To explore the molecule mechanism of YM155 treatment, we try to explore new target and “net work” of YM155 with a powerful platform, Ingenuity Pathway Analysis program.

The basis of the IPA program consists of the Ingenuity Pathway Knowledge Base (IPKB) which is derived from known functions and interactions of genes published in the literature. The IPA Tool allows the identification of biological networks, global functions and functional pathways of a particular dataset. The program also gives the significance value of the genes, the other genes with which it interacts, and how the products of the genes directly or indirectly act on each other, including those not involved in the microarray analysis. This work represents cell death was the highest rated network with 65 focus molecules and the significance score of 44. Death receptor signaling, TNFR1 signaling ,induction of apoptosis by HIV1 ,apoptosis signaling and molecular mechanisms of cancer came out to be the top four most significant pathways. IPA analysis also showed top molecules up-regulated was BBC3 (PUMA). PUMA encodes a member of the BCL-2 family of proteins. This family member belongs to the BH3-only pro-apoptotic subclass. The protein cooperates with direct activator proteins to induce mitochondrial outer membrane permeabilization and apoptosis. It can bind to anti-apoptotic Bcl-2 family members to induce mitochondrial dysfunction and caspase activation. Because of its pro-apoptotic role, this gene is a potential drug target for cancer therapy and for tissue injury. IPA analysis also showed upstream regulators were NR3C1, TP53, dexamethasone, TNF and Akt. These upstream regulators such as TP53, TNF and Akt have already been reported as important regulators for the survivin network. TP53 and Akt have been widely investigated and there are hundreds of papers about the important roles in survivin pathway. But there is still no report about the relationship between NR3C1, dexamethasone and survivin. NR3C1 gene encodes glucocorticoid receptor, which can function both as a transcription factor that binds to glucocorticoid response elements in the promoters of glucocorticoid responsive genes to activate their transcription and as a regulator of other transcription factors. This receptor is typically found in the cytoplasm, but upon ligand binding, is transported into the nucleus. It is involved in inflammatory responses, cellular proliferation, and differentiation in target tissues. Dexamethasone gene encodes a member of the Ras superfamily of small GTPases and is induced by dexamethasone. The encoded protein is an activator of G-protein signaling and acts as a direct nucleotide exchange factor for Gi-Go proteins. This protein interacts with the neuronal nitric oxide adaptor protein CAPON, and a nuclear adaptor protein FE65, which interacts with the Alzheimer's disease amyloid precursor protein. This gene may play a role in dexamethasone-induced alterations in cell morphology, growth and cell-extracellular matrix interactions. Epigenetic inactivation of this gene is closely correlated with resistance to dexamethasone in multiple myeloma cells. This work indicated firstly that NR3C1and dexamethasone may be upstream regulators in the survivin pathway. These results may provide new clues of molecular mechanism of apoptosis induced by YM155.

## Conclusions

The present study demonstrates that YM155 treatment resulted in apoptosis and inhibition of cell proliferation of SK-NEP-1cells. YM155 had significant role and little side effect in the treatment of SK-NEP-1 xenograft tumors. Real-time PCR array analysis firstly showed expression profile of genes dyes-regulated after YM155 treatment. IPA analysis also represents new molecule mechanism of YM155 treatment, such as NR3C1 and dexamethasone may be new target of YM155. And our results may provide new clues of molecular mechanism of apoptosis induced by YM155.

## Competing interests

The authors have no conflicts of interest to disclose.

## Authors’ contributions

PJ designed and directed the study. WJ drafted the manuscript. TYF and LJ finished the most of the experiments. FX, ZX, PL, CL, XPF, PL and LYH coordinated data collection and quality control, and assisted in the interpretation of results. DXJ, SLC, WD and WN participated in acquiring laboratory data analysis. NJ participated in study design and coordination, data analysis and interpretation and drafted the manuscript. All authors read and approved the final manuscript.

## Pre-publication history

The pre-publication history for this paper can be accessed here:

http://www.biomedcentral.com/1471-2407/12/619/prepub

## Supplementary Material

Additional file 1 Cluster analysis the data from Real-time PCR arrays. Click here for file

Additional file 2 Summary of IPA analysis. Click here for file
