# Supplementary figures and images for: Survivin selective inhibitor YM155 induce apoptosis in SK-NEP-1 Wilms tumor cells
Source: BMC Cancer. 2012 Dec 26;12:619. doi: 10.1186/1471-2407-12-619 (PMC3543843; doi:10.1186/1471-2407-12-619)

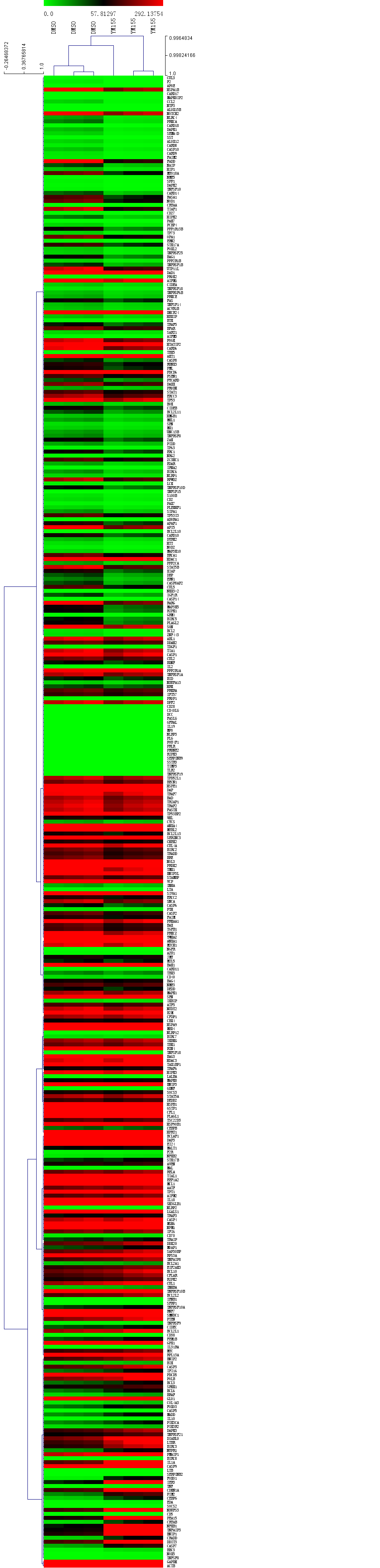

Supplement: Additional file 1 — Cluster analysis the data from Real-time PCR arrays. [file 1471-2407-12-619-S1.tiff]
