# Supplementary material for: Survivin selective inhibitor YM155 induce apoptosis in SK-NEP-1 Wilms tumor cells
Source: BMC Cancer. 2012 Dec 26;12:619. doi: 10.1186/1471-2407-12-619 (PMC3543843; doi:10.1186/1471-2407-12-619)
Supplement: Additional file 2 — Summary of IPA analysis. [file 1471-2407-12-619-S2.pdf]

IPA® Summary of Analysis

Analysis Name: API-YM155 - 2012-08-16 09:14
Analysis Creation Date: 2012-08-16
Build version: 162830
Content version: 12710793 (Release Date: 2012-05-07)

Analysis settings

View

Reference set: Ingenuity Knowledge Base (Genes Only)

Relationship to include: Direct and Indirect

Includes Endogenous Chemicals

Optional Analyses: My Pathways My List

Filter Summary:

Consider only molecules and/or relationships where

(species = Uncategorized (e.g. chemicals) OR Human) AND

(confidence = Experimentally Observed) AND

(tissues/cell lines = Effector memory helper T cells OR Effector memory cytotoxic T cells OR Vd1 Gamma-delta T cells OR RAW 264.7 OR Activated Vd1 Gamma-delta T cells OR Macrophages OR Activated CD56bright NK cells OR Monocytes OR MOLT-4 OR Other Tissues and Primary Cells OR Prostate Gland OR Effector T cells OR Activated CD56dim NK cells OR SR OR Large Intestine OR Murine NKT cells OR Memory B cells OR Heart OR Natural T-regulatory cells OR Activated Vd2 Gamma-delta T cells OR Kidney OR Naive B cells OR Jurkat OR Plasmacytoid dendritic cells OR Adipose OR CD56bright NK cells OR Mature monocyte-derived dendritic cells OR Monocyte-derived macrophage OR Naive helper T cells OR CD56dim NK cells OR Effector memory RA+ cytotoxic T cells OR Thymus OR Liver OR HL-60 OR Lung OR Activated helper T cells OR Bladder OR Uterus OR Immature monocyte-derived dendritic cells OR BDCA-1+ dendritic cells OR Ovary OR Placenta OR Skeletal Muscle OR THP-1 OR Small Intestine OR BDCA-3+ dendritic cells OR Retina OR Central memory cytotoxic T cells OR

(c) 2000-2012 Ingenuity Systems, Inc. All rights reserved.

Neutrophils OR Spleen OR Vd2 Gamma-delta T cells OR Salivary Gland OR Stomach OR Cytotoxic T cells OR Epidermis OR Th1 cells OR Central memory helper T cells OR CCRF-CEM OR Th2 cells OR K-562 OR Mammary Gland OR Pancreas OR Testis) AND
(data sources = BIND OR BIOGRID OR Cognia OR DIP OR Ingenuity Expert Findings OR Ingenuity ExpertAssist Findings OR INTACT OR Interactome studies OR MINT OR MIPS OR TarBase)

Top Networks

ID	Associated Network Functions	Score
1	Cell Death, Embryonic Development, Renal Necrosis/Cell Death	44
2	Cell Death, Tumor Morphology, Cellular Development	25
3	Cell Death, Ophthalmic Disease, Developmental Disorder	24
4	Cell Death, Inflammatory Response, Antigen Presentation	20
5	Cell Cycle, Cellular Development, Cellular Growth and Proliferation	18

(c) 2000-2012 Ingenuity Systems, Inc. All rights reserved.

INGENUITY® SYSTEMS

Top Bio Functions

Diseases and Disorders

Name	p-value	# Molecules
Cancer	2.51E-09 - 6.27E-03	51
Hematological Disease	2.51E-09 - 6.27E-03	22
Gastrointestinal Disease	4.92E-08 - 6.27E-03	24
Hepatic System Disease	4.92E-08 - 6.27E-03	12
Inflammatory Disease	4.92E-08 - 6.27E-03	23

Molecular and Cellular Functions

Name	p-value	# Molecules
Cell Death	5.43E-37 - 6.27E-03	65
Cellular Function and Maintenance	3.07E-11 - 6.27E-03	30
Cell Morphology	4.69E-11 - 6.27E-03	22
Carbohydrate Metabolism	1.27E-10 - 6.27E-03	14
Cellular Growth and Proliferation	1.55E-10 - 6.27E-03	32

Physiological System Development and Function

Name	p-value	# Molecules
Embryonic Development	3.75E-17 - 6.27E-03	23
Tumor Morphology	1.29E-06 - 6.27E-03	12
Tissue Development	1.60E-05 - 6.27E-03	15
Tissue Morphology	1.65E-05 - 4.41E-03	8
Hematological System Development and Function	1.95E-05 - 6.27E-03	16

(c) 2000-2012 Ingenuity Systems, Inc. All rights reserved.

Top Canonical Pathways

Name	p-value	Ratio
Death Receptor Signaling	3.87E-19	14/63 (0.222)
TNFR1 Signaling	2.34E-13	10/52 (0.192)
Induction of Apoptosis by HIV1	2.67E-12	10/64 (0.156)
Apoptosis Signaling	6.56E-12	11/94 (0.117)
Molecular Mechanisms of Cancer	2.13E-11	17/371 (0.046)

Top Molecules

Fold Change up-regulated

Molecules	Exp. Value	Exp. Chart
BBC3	1.000	
BIRC3	1.000	
BIRC8	1.000	
BNIP1	1.000	
CASP7	1.000	
CASP9 (includes EG:100140945)	1.000	
CD5	1.000	
CDKN1A	1.000	
CEBPG	1.000	
COL4A3	1.000	

Fold Change down-regulated

Molecules	Exp. Value	Exp. Chart
ZNF443	-1.000	
UTP11L	-1.000	
TP73	-1.000	

(c) 2000-2012 Ingenuity Systems, Inc. All rights reserved.

INGENUITY® SYSTEMS

TNFSF10	-1.000
TNFRSF1B	-1.000
TNFRSF25	-1.000
TIAF1	-1.000
STK17A	-1.000
SST	-1.000
SPP1 (includes EG:20750)	-1.000

Top Upstream Regulators

Upstream Regulator	p-value of overlap	Predicted Activation State
NR3C1	1.99E-24	Inhibited
TP53 (includes EG:22059)	3.37E-16	
dexamethasone	1.65E-13	
TNF	3.44E-13	Activated
Akt	3.90E-13	

(c) 2000-2012 Ingenuity Systems, Inc. All rights reserved.

Top My Lists

Name	p-value	Ratio
BIOMARKERS HNC and mouse HPV16	4.65E-01	1/100 (0.01)

Top My Pathways

Name	p-value	Ratio
IL24 pic	3.36E-03	3/54 (0.056)
FASN malignancy pathway	1.84E-02	3/143 (0.021)
Merged networks	8.23E-02	2/268 (0.007)
NRF2-mediated Oxidative Stress Response	2.89E-01	1/84 (0.012)

Top Tox Lists

Name	p-value	Ratio
Renal Necrosis/Cell Death	2.42E-18	24/368 (0.065)
Pro-Apoptosis	6.83E-14	10/42 (0.238)
Anti-Apoptosis	2.43E-13	9/32 (0.281)
Liver Necrosis/Cell Death	1.26E-11	15/232 (0.065)
Decreases Transmembrane Potential of Mitochondria and Mitochondrial Membrane	1.55E-11	11/95 (0.116)

(c) 2000-2012 Ingenuity Systems, Inc. All rights reserved.

INGENUITY® SYSTEMS

Top Tox Functions

Assays: Clinical Chemistry and Hematology

Name	p-value	# Molecules
Increased Levels of CRP	1.25E-02 - 1.25E-02	1
Increased Levels of LDH	4.31E-02 - 4.31E-02	1

Cardiotoxicity

Name	p-value	# Molecules
Cardiac Necrosis/Cell Death	1.25E-03 - 6.10E-02	4
Cardiac Damage	6.27E-03 - 6.27E-03	1
Cardiac Hypertrophy	1.25E-02 - 4.36E-02	3
Cardiac Inflammation	1.25E-02 - 1.25E-02	1
Cardiac Stress Response	1.25E-02 - 1.25E-02	1

Hepatotoxicity

Name	p-value	# Molecules
Liver Necrosis/Cell Death	3.16E-08 - 6.69E-02	7
Liver Cirrhosis	3.73E-05 - 1.35E-04	7
Liver Proliferation	3.89E-05 - 2.48E-02	4
Hepatocellular Carcinoma	3.87E-03 - 1.15E-02	8
Liver Hyperplasia/Hyperproliferation	3.87E-03 - 1.15E-02	8

Nephrotoxicity

Name	p-value	# Molecules
Renal Necrosis/Cell Death	2.42E-17 - 5.51E-02	23
Renal Proliferation	4.96E-04 - 1.49E-01	4
Glomerular Injury	6.27E-03 - 6.27E-03	1

(c) 2000-2012 Ingenuity Systems, Inc. All rights reserved.

Renal Inflammation	1.25E-02 - 3.93E-01	3
Renal Nephritis	1.25E-02 - 3.93E-01	3

(c) 2000-2012 Ingenuity Systems, Inc. All rights reserved.

INGENUITY SYSTEMS
